# Inclusion of pyridoxine to flaxseed cake in poultry feed improves productivity of omega-3 enriched eggs

**DOI:** 10.6026/97320630015333

**Published:** 2019-04-30

**Authors:** Shahida Aziz Khan

**Affiliations:** 1 Applied Nutrition Group, King Fahd Medical Research Center, King Abdulaziz University, Jeddah, Kingdom of Saudi Arabia; 2 Department of Medical Laboratory Technology, Faculty of Applied Medical Sciences, King Abdulaziz University, Jeddah, Kingdom of Saudi Arabia

**Keywords:** Flaxseed cake, anti-nutrients, pyridoxine, poultry feed

## Abstract

Flaxseed cake produced during flax oil production is rich in protein, and could be utilized in the production of omega-3 enriched eggs or value added eggs. But the presence of anti-nutrients in flaxseed cake adversely affects the bird and egg characteristics. Supplementation of pyridoxine may prove beneficial as noted by earlier research. In our study, we divided 30 layer birds into three groups and supplemented them with different combination feeds containing: (A) control ration, (B) fresh flax seed cake with pyridoxine and (C) fresh flax seed cake without pyridoxine. Crushed 2% flaxseed was also added to the flaxseed cake groups for deposition of sufficient omega-3 fatty acids into the eggs. Supplementation was given for a period of 4 weeks and egg as well as bird characteristics observed. Birds fed pyridoxine showed better incorporation of omega-3 fatty acids into the egg yolk. Supplementation of flaxseed cake with pyridoxine resulted in better yields of omega-3 eggs as well as improved health of layer birds

## Background

Omega-3 fatty acids play an important role in controlling the inflammatory processes of the body that result in chronic conditions such as high blood pressure, type 2 diabetes, coronary heart disease etc. 1, 2. Omega-3 enriched eggs are a good source of omega-3 fatty acids in a natural edible form. Flaxseed is a very rich source of the parent omega-3 fatty acid, namely alpha-linolenic acid, and flaxseed oil contains 45-52% of this fatty acid 3. The seed, oil and cake could well be used for the production of omega-3 enriched eggs. Flaxseed cake (FC) produced during flax oil production is also an excellent protein source for livestock 4, 5, containing between 10.5% and 31% of protein 6. It contains residual oil that is rich in alpha linolenic acid, which positively contributes to good animal health 7. FC contains about 1% of residual oil, extendable up to around 16%, depending upon the seed strain, and processing conditions 8. This makes FC a potential candidate for production of omega-3 eggs, and increasing the deposition of omega-3 fatty acids into the bird egg yolk 9. However, a 15% FC supplementation to layer birds showed a lower deposition of linolenic acid as compared to 15% flaxseed 10. This is because of the fact that apart from the immense benefits, flaxseeds are known to contain certain anti-nutritional factors, 11 like cyanogenic glycosides, which get concentrated in the FC. A decrease in bioavailability affecting the growth, feed intake, egg characteristics and hen physiology has been earlier noted when the FC was incorporated in poultry feeds. 3. The presence of the compounds linatine and 1-amino-D-proline render poultry diets containing FC to become deficient in pyridoxine (vitamin B6) as they exhibit antagonistic activity towards the vitamin 12. A decrease in egg production along with a decrease in bird weight has been observed for layer birds fed 30% of flaxseed cake as compared to birds fed on a 10 %, and 20% flaxseed cake supplemented diet 13. Feeding FC in concentrations of 15 and 22.5% has been found to reduce total nutrient digestibility as well as energy utilization, but 7.5% could retain the egg characteristics 14. Also the presence of high mucilage in FC leads to the formation of a gummy substance that suppresses the hen�s eating ability 15. Larger amounts of FC in feed are hence known to suppress growth and development of the birds. Feeding flaxseed at 10%concentration has been found to cause an increased damage to the liver 16. The damage is due to the liver containing high levels of long chain unsaturated fatty acids 17 that are very prone to lipid peroxidation. Addition of flaxseed is known to cause an increase in serum glutamic oxaloacetic transaminase (SGOT), and serum glutamic pyruvic transaminase (SGPT) blood levels due to physiological stress 18 to the bird. The use of FC is hence not very popular in the production of eggs, and its incorporation into food products raises issues of safety, and quality control 19. Due to all this, reducing the toxicity has been an ever-growing challenge with newer combinations and economical feeds being tried out. The threshold of FC recommended is between 3-10 % to avoid any toxic effects to poultry 20. FC stored for three months was fairly stable with peroxide values not exceeding the threshold of 10 meq per Kg. Also procedures to minimize the anti-nutrients could be done using earlier methods tried out 13. One of the methods of correcting this anti-nutrient ability was suggested to be the addition of vitamin B6 to poultry feed 21. Earlier research has shown that supplementing 40mg/kg of vitamin B6 to the poultry diet-containing 140g per Kg of ground flaxseed did not exhibit any alterations in the bird characteristics 22. However, the influence of lower concentrations of flaxseed cake on transfer of fatty acids into the egg yolk of layer birds is yet to be elucidated further. Therefore we tried to maximize the usage of FC, and negate the toxic effects, by supplementing minimal quantities of vitamin B6 to feeds with 5% FC containing residual flaxseed oil, to enhance omega-3 egg production and assess its role on egg and layer bird characteristics.

## Methodology

### Experimental Plan for feed, FC selection and storage:

Moisture content of the FC was maintained well below 9% (analyzed using oven drying and weighing method) throughout the course of this study. FC was stored under nitrogen gas (passed through a tube and immediately container sealed) at 4 degree Celsius to avoid moisture, and prevent oxidation. Also fresh and mature flaxseeds were crushed under ideal conditions i.e. reasonably high temperature and moisture free environment to prevent prussic acid toxicity 15. 5% flaxseed cake of the first extracted oil batch containing around 7% of residual oil was utilized in the study. Vitamin B6 was additionally supplemented to ensure safety to the bird as well as for the efficient production of omega-3 enriched eggs.

Supplemental feed rich in omega-3 fatty acids, and other nutrients was provided by our institute to the poultry to feed layer hens (age 20 Weeks). The diets were kept refrigerated throughout the experiment, until feed was due. A total of 30 layer birds (Hy Line w-36) were kept in pens and the room maintained at 25°C. Two hens housed in 1 experimental cage of size (38 - 41 cm) were considered as 1 replicate. Five replications were made for each diet. Water and feed were provided ad libitum. The experimental diets were fed for a period of 4 weeks. Birds were serially numbered and fed with supplemental feed provided, after taking into consideration the appropriate weight deductions from control ration. Every week on the 5th day of production feed was supplied, as well as eggs collected. Bird and egg characteristics were subsequently analyzed and recorded. Diets were provided as follows: (1) Group A (control)----10 birds fed normal control ration only; (2) Group B ---10 birds fed 2% flaxseed crushed + 5% FC +Vitamin B6 (40mg/Kg) along with control ration; (3) Group C-- 10 birds fed 2% flax seed crushed, +5% FC, along with control ration. 2% crushed flaxseed was added to the diets for the incorporation of sufficient omega-3 fatty acids into the egg yolks.

### Cholesterol estimation in egg yolk:

Estimation of cholesterol in egg yolk was carried out by Atozyme cholesterol enzymatic kit using the method of Kishi 23.

### Fatty acid analysis in egg yolk:

Methyl esters of the yolk fractions were prepared and quantified 24 using an Agilent model 7890 gas chromatograph. A fused silica gel Supelco USA column with 30 m length, 0.32 mm inner diameter and 0.25 μm thickness was used.

### Bird blood analysis:

An independent private laboratory performed Blood lipid profile, SGOT and SGPT tests.

## Results

### Egg and bird characteristics

In our study we were able to effectively raise the omega -3 content of the egg apart from the protein enrichment by flaxseed cake, without any adverse outcomes on egg and bird characteristics. It was observed that the mucilage present in FC caused sticky droppings, but this did not affect the layer bird characteristics. No mortality was observed in all the groups throughout the study period. Enhancement in feed consumption was similar in all the groups studied from week zero to week four with a slight reduction in group C which was supplemented only with FC. Egg parameters showed improvements in the group fed with vitamin B6. Egg weights increased with time in all the three groups studied. Shell thickness and shell weight was significantly increased (p value 0.000) in group B supplemented with vitamin B6 as compared to the other two groups as seen in Table 1.

Though albumen weights increased significantly in all the three groups, only the two groups supplemented with FC showed significant enhancement in yolk weights. Also yolk albumen ratio was found enhanced significantly (p value 0.033) only in the group supplemented with vitamin B6 (Table 2). Feeding FC based diets with vitamin B6 resulted in positive effects on egg production characteristics when compared with the control diet, by not only minimizing the adverse effects of FC supplementation but also improving productivity. Cholesterol analysis of egg yolk showed that cholesterol levels were lowered significantly (p value 0.000) in Groups B, Groups B & C when compared to the control group A after a period of four weeks (Table 2).

GC analysis showed that total omega - 3 fatty acid content in the egg yolk of groups B & C enhanced significantly (p value 0.000) as compared to the control group A as observed in Table 3. Total omega-6 content was reduced in groups B & C but the group B supplemented with vitamin B6 showed significant reduction with a p value of 0.005. Omega-6/omega-3 ratio was found to be significantly lower in both groups B and C which contain only FC with a p value of 0.000 in both. The control group A did not show any appreciable changes in the omega-6/omega-3 ratio and showed the highest ratio.

### Bird blood analysis:

Triglycerides showed a reduction in groups B & C, but only the B group containing vitamin B6 showed significance with a p value 0.016. Lipid profile improved with the addition of FC and was further improved with vitamin B6 inclusion as observed in Table 4. Total blood cholesterol showed a significant decrease in groups B and C, while the value slightly increased in the control group. A decrease in cholesterol in groups B & C is observed, which can be attributed to the increased amount of omega-3 fatty acid in both the groups. Values of the healthy cholesterol HDL improved significantly in both groups B (p value 0.002) & C (p value 0.005), with the B group showing greater significance. Values of LDL cholesterol remained almost the same in the control group, but decreased significantly in groups B and C with p values 0.000 and 0.001 respectively.

Serum glutamic oxaloacetic transaminase (SGOT) increased significantly in all the groups but showed significant increases in the groups A and C. Group B exhibited a lower p value of 0.012 showing the protective ability of vitamin B6as compared to 0.000 in the A&C group. Serum glutamic pyruvic transaminase (SGPT) levels showed only a slight increase in the control group A while group C supplemented only with FC exhibited stress on the liver showing a significant increase with a p value of 0.047. But SGPT levels in group B were reduced significantly with a p value of 0.000 as observed in Table 4.

## Discussion

A successful attempt in combating the anti-nutritional effects of flaxseed could be done, by including pyridoxine along with 5% FC in poultry feed. As our experiment was done for a period of only four weeks, we did not observe any appreciable changes in feed intakes. A continuous feed intake of anti-nutrients in flax meal for a longer period of time may be attributed to the lower levels observed by earlier researchers 16. Earlier researchers have noted altered palatability; thereby reducing feed consumption 25. But in our experiment, we could overcome this problem by the addition of health promoting nutrient like vitamin B6 in appropriate quantities.

The FC diet showed a positive effect on egg cholesterol lowering due to the presence of alpha-linolenic acid. A linear increase in omega6/omega3 ratio is observed in the groups, with lowest ratio in the group B supplemented with vitamin B6 and highest in the control group. Therefore, it seems that vitamin B6 in the diet may have an effect on desaturases in laying birds, resulting in alterations of egg fatty acid concentrations.

Serum lipid profile showed an improvement with lowering of triglycerides, total cholesterol, and LDL with a concomitant increase in the good cholesterol HDL. This is in accordance with earlier research wherein improvements in growth and lipid values were observed 26. As the levels of SGOT and SGPT are reasonable indicators of damage or injury to the liver, it is necessary to lower them for efficient conversion. Uptake of the omega-3 fatty acid without causing excess stress on the liver, is importantly beneficial in the production of omega-3 enriched eggs, whilst lowering the SGOT and SGPT levels 27. This would not only enhance the bird safety but also boost omega-3 egg production and reduce bird stress, thereby improving sales. In our study we found decreases in the SGPT levels only in group B, which is quite encouraging. Nevertheless a long-term study may give us a better understanding on the stress effects on the liver.

## Conclusion

Anti-nutrients suppressing the growth and egg laying ability in layer birds have been noted in the past. In our study we could utilize the nutrients of flaxseed cake without any adverse effects. FC (5%) could thus be used successfully in the prevalent climatic conditions of KSA by supplementing adequate levels of vitamin B6 to counteract its anti-metabolite present in FC. Long term studies using vitamin B6, encompassing the entire egg laying period need to be done to study economic feasibility. FC being cheaply available could thus be put to better usage with appropriate inclusions. Also such production of omega-3 fatty acid and vitamin enriched eggs through cheaper sources, would give poultry farmers an opportunity to be part of an emerging industry that would not only increase consumer acceptance but also good economic returns.

## Conflict of Interest

Authors declare no conflict of interest.

## Figures and Tables

**Table 1:** Physical characteristics of egg and layer birds

		Control (A)	With B6 (B)	Without B6 (C)
Feed consumption (gm)	0wk	104.910± 1.516	105.185± 1.861	104.150±1.312
	4thwk	113.529±1.820	111.212± 1.972	107.485±2.210
	p value	0.000***	0	0.003**
Mortality (No.)	0wk	0	0	0
	4thwk	0	0	0
	p value	0	0	0
Egg weight (gm)	0wk	45.384 ± 0.794	44.935 ± 0.979	45.175±0.677
	4thwk	47.251 ±0.466	48.630±1.110	47.664 ± 0.920
	p value	0.000***	0.000***	0.000***
Egg shell weight (gm)	0wk	4.466± 0.331	4.606± 0.249	4.584± 0.206
	4thwk	4.634±0.350	5.023±0.106	4.629± 0.280
	p value	0.273	0.003**	0.692
Egg shell thickness (mm)	0wk	0.326±0.016	0.323± 0.013	0.320± 0.014
	4thwk	0.324± 0.015	0.349± 0.012	0.325± 0.014
	p value	0.443	0.000***	0.299

**Table 2:** Yolk, Albumen, and Cholesterol content in eggs

		Control(A)	With B6 (B)	Without B6 (C)
No.of birds		10	10	10
Yolk weight (gm)	0wk	13.793± 0.693	13.625± 0.452	13.741± 0.512
	4thwk	14.138± 1.173	15.246± 0.547	14.768± 0.342
	p value	0.356	0.000***	0.000***
Albumen weight (gm)	0wk	27.089± 1.025	27.032±1.483	26.664± 1.129
	4thwk	28.028± 1.236	28.240± 1.383	28.226± 1.142
	p value	0.001**	0.012*	0.024*
Yolk/Albumen ratio	0wk	0.510 ±0.042	0.505± 0.038	0.516± 0.036
	4thwk	0.507± 0.067	0.540± 0.043	0.524± 0.031
	p value	0.841	0.033*	0.627
Egg cholesterol	0wk	200.865± 15.090	166.605± 4.916	180.319± 6.232
	4thwk	205.222± 29.471	109.680± 2.308	132.969± 9.404
	p value	0.648	0.000***	0.000***

**Table 3:** Fatty acid percent in eggs produced

		Control (A)	With B6 (B)	Without B6 (C)
Triglycerides (mg/dl)	0 wk	140.460 ± 15.426	128.240 ± 22.855	168.000± 59.908
	4thwk	134.230± 16.689	105.773± 5.585	127.076± 31.130
	p value	0.367	0.016*	0.089
Total cholesterol (mg/dl)	0 wk	147.500 ± 22.653	144.522 ± 10.072	139.243 ± 12.732
	4thwk	153.900 ± 13.379	103.128± 5.736	118.270 ± 15.924
	p value	0.564	0.000***	0.002**
HDL (mg/dl)	0 wk	69.349± 2.329	69.649± 3.900	72.363 ± 3.770
	4thwk	68.273 ± 2.474	76.466± 2.649	73.504 ± 3.247
	p value	0.096	0.002**	0.005**
LDL (mg/dl)	0 wk	79.880 ± 8.287	44.304 ± 3.766	42.358 ± 4.754
	4thwk	81.180 ± 11.379	31.927 ± 3.485	32.581 ± 4.230
	p value	0.641	0.000***	0.001**
SGOT (IU/L)	0 wk	173.700 ± 16.734	210.800 ± 51.549	166.200 ± 36.475
	4thwk	233.900 ± 36.263	258.700 ± 18.099	266.300 ± 38.134
	p value	0.001	0.012*	0.000**
SGPT (IU/L)	0 wk	14.570 ± 1.497	13.720 ± 2.413	13.400 ± 3.340
	4thwk	15.270 ± 1.585	6.210 ± 1.018	15.930 ± 1.463
	p value	0.177	0.000***	0.047*

**Table 4:** Bird blood characteristics

		Control(A)	With B6(B)	Without B6(C)
No. of birds		10	10	10
Total percent omega-3 fatty acids	0 wk	0.79 ± 0.127	1.158 ± 0.267	0.950 ± 0.461
	4thwk	1.163 0.418	2.327 ± 0.284	1.995± 0.304
	p value	0.1093	0.000***	0.000***
Total percent omega-6 fatty acids	0 wk	7.933 ± 3.207	11.982 ± 2.406	10.612± 2.864
	4thwk	10.511 ± 3.676	9.150 ± 0.396	9.380 ± 0.893
	p value	0.0947	0.005**	0.215
Omega-6/omega-3 fatty acid ratio	0 wk	10.029± 3.893	10.713 ± 2.948	12.251 ± 3.747
	4thwk	9.066± 0.083	3.967 ± 0.330	4.747 ± 0.444
	p value	0.9232	0.000***	0.000***

## References

[1] Simopoulos 
AP (2006). Biomed Pharmacother.

[2] Connor 
WE (2000). Am J ClinNutr.

[3] Faqir  MA
 (2013). Lipids in Health and Disease..

[4] Gutierrez  C
 (2010). Soil Sci Plant Nutr..

[5] Hall  C (2006). Advances in food and nutrition research in Flaxseed..

[6] Shim YY (2014). Trends in Food Science and Technology.

[7] Olomu JM, Baracos VE ( 1991). Poultry Sci.

[8] Shim  YY (2015). Trends in Food Science Technology..

[9] Nain S (2012). Poult Sci..

[10] Jia W (2008). Poult Sci..

[11] Krishna B (2015). Int J Pharm Sci Rev Res..

[12] Mayengbam 
S (2014). Journal of Agricultural and Food Chemistry..

[13] Imran 
M  (2015). Lipids in health and disease..

[14] Huang 
S (2018). British Poultry Science.

[15] Kajla 
P (2015). J Food Sci Technol..

[16] Bean 
LD, Leeson S (2003). PoulSci..

[17] Caston LJ (1994). Canadian Journal of Animal Science..

[18] Sahini 
K (2002). Vet Med � Czech..

[19] Goyal 
A (2014). J Food Sci Technol..

[20] https://flaxcouncil.ca/wp-content/uploads/2015/02/Flax-Feed-Industry-Guide-Final.pdf.

[21] Daghir 
NJ (1976). Worlds Poultry Science Journal..

[22] Shen 
Y, Chavez RE ( 2003). J Sci Food Agric.

[23] Kishi 
OK (2002). Clin Chem..

[24] Masood 
A  ( 2005). J Lipid Res..

[25] Rodr�guez 
ML ( 2001). British Poultry Science..

[26] Ali 
HA ( 2018 ). Pakistan Journal of Nutrition..

[27] Sara 
AM (. 2016). Scientific Research Journal (SCIRJ).

